# An Ionically Conductive, Self-Powered and Stable Organogel for Pressure Sensing

**DOI:** 10.3390/nano12040714

**Published:** 2022-02-21

**Authors:** Li Wang, Zhengduo Wang, Yingtao Li, Yu Luo, Bingheng Lu, Yiyang Gao, Wei Yu, Guoxin Gao, Shujiang Ding

**Affiliations:** 1 Micro and Nano Technology Research Center, State Key Laboratory for Manufacturing Systems Engineering, Xi’an Jiaotong University, Xi’an 710049, China; wangzhengduo@stu.xjtu.edu.cn (Z.W.); liyingtao_lubu@163.com (Y.L.); yuluo825@mail.xjtu.edu.cn (Y.L.); bhlu@mail.xjtu.edu.cn (B.L.); 2 National Innovation Institute of Additive Manufacturing, Xi’an 710000, China; 3 Xi’an Key Laboratory of Sustainable Energy Materials Chemistry, School of Chemistry, Xi’an Jiaotong University, Xi’an 710049, China; yilimengniu0616@stu.xjtu.edu.cn (Y.G.); yuwei2019@mail.xjtu.edu.cn (W.Y.); gaoguoxin@mail.xjtu.edu.cn (G.G.); dingsj@mail.xjtu.edu.cn (S.D.)

**Keywords:** organogel, self-powered, stable, pressure sensing

## Abstract

Gel-based ionic conductors are promising candidates for flexible electronics, serving as stretchable sensors or electrodes. However, most of them suffer from a short operating life, low conductivity and rely on an external power supply, limiting their practical application. Herein, we report a stable organogel ionic conductor with high conductivity and self-powering ability. Briefly, lithium trifluoromethanesulfonate, as a conductive salt, provides high conductivity and the poly(1,1-difluoroethylene) layers, as a self-powering system, supply stable energy output under the influence of pressure. Moreover, the proposed conductors withstand long-term and multi-cycle durability tests. The prepared auxiliary training device can withstand the impact of a basketball and detect the impact force, showing potential in passive sensing during practical applications.

## 1. Introduction

The properties of being able to closely fit the complex surface of an object, with strong anti-bending abilities and high electrical conductivity are important for flexible electronics [1,2,3,4], such as soft sensors [5,6,7], flexible displays [8,9], and wearable computing [10,11,12] and energy storage devices [13,14,15]. To achieve these characteristics, researchers have developed various materials and structures, such as nanocomposites [16,17], bionic structures [18,19,20], and sensor arrays [21,22]. However, the inherent high modulus and brittleness lead to an unacceptable mechanical mismatch [23,24] and the unsatisfying electrical conductivity [25,26] results in inevitable signal loss during transmission. Liu et al. have integrated perovskite solar cells (PSCs) into flexible strain sensors to realize continuous data recording without external power [27]. Although the high efficiency of PSC (8.41%) resulted in excellent stability and durability, the mismatch between tissues and PET substrate hinders the wearable applications of these sensors.

With Young’s modulus values close to tissues, gels are the most promising candidates for flexible devices [2,28,29]. However, the conductivity of gels is not enough for their direct application in electronic devices [30]. Hence, the most effective method to enhance the conductivity is to add conductive fillers [31,32,33,34]. For instance, Yunsik Ohm et al. incorporated silver flakes (5 vol%) into the hydrogels and performed a partial dehydration process to remove a moderate amount of water and form the percolation channels [1]. Although silver-containing hydrogels possess high conductivity (350 S·cm^−1^), the silver flakes reduce stretchability, increase the Young’s modulus and generate massive Joule heat under high currents, leading to catastrophic water loss and accelerating the gels’ invalidation.

Researchers have explored numerous methods to overcome water loss, such as adding anti-freeze materials (e.g., polyelectrolytes, polyhydric alcohols) [35,36] or encapsulation [37,38]. Anti-freeze materials reduce the number of hydrogen bonds between water molecules and block the gel from freezing; however, water evaporation remains a challenge [39,40]. On the other hand, the packaging methods lock the water inside the container and reduce water evaporation; however, these methods are not suitable for freezing and boiling [6,41]. Therefore, organic ionic gels have been developed, which essentially circumvent the problems of hydrogels as organic solvents [42]. However, the high viscosity of organic gels hinders ionic movement and, in turn, reduces the conductivity [43]. Therefore, it is necessary to explore different approaches to enhance the conductivity of organic ionic gels.

Herein, we report high-conductivity organogel ionic conductors (OICs) and their piezoelectrically-enhanced analogs, using a one-step synthesis method, where propylene carbonate (PC), acryloyl morpholine (ACMO) and lithium trifluoromethanesulfonate (LiOTf) are employed as a solvent, monomer and ionically conductive salt, respectively. The as-prepared OICs exhibit high ionic conductivity (9.1 × 10^−4^ S·cm^−1^) and a wide range of temperature tolerances (−70 to 100 °C). Moreover, OICs are further coated with poly(1,1-difluoroethylene) (PVDF) as the power supply. Owing to the penetration effect, the number of β-type crystals generated by PVDF on the OICs was found to be 70% higher than that on a glass substrate (40%). Furthermore, the self-powered OICs maintained high accuracy even after one month of storage, demonstrating broad application prospects as self-powered flexible conductors and sensors.

## 2. Materials and Methods

### 2.1. Materials

Propylene carbonate (PC), 4-acryloylmorpholine (ACMO, monomer), poly-ethylene-glycol diacrylate (PEGDA Mw~1000), 1-hydroxycyclohexyl phenyl ketone (184), lithium trifluoromethanesulfonate (LiOTf), N,N-dimethylformamide (DMF), Sudan III and poly(1,1-difluoroethylene) (PVDF) were purchased from Aladdin, Co. Ltd., Shanghai, China, and the silk fibroin (SF) was synthesized by our research team.

### 2.2. Preparation of OICs and Self-Powered OICs

The preparation process is summarized below [42]: PEGDA and 184 were employed as a cross-linker and photo-initiator, respectively. First, LiOTf, 184, ACMO (33 wt.%) and PEGDA were evenly dissolved in PC (64 wt.%) to create a transparent precursor of OICs under mechanical stirring for at least 15 min or 5 min of ultrasonication. The concentration of LiOTf was adjusted to 2.5 mol·L^−1^, and PEGDA was fixed at 0.1 wt.%. Subsequently, the precursors were cured in a glass-wrapped polyethylene glycol terephthalate (PET) mold under irradiation of a 365 nm ultraviolet lamp for 5 min to obtain OICs. To obtain self-powered OICs, 1 g of PVDF was dissolved in 8 mL of DMF at 90 °C for 8 h to obtain a uniform solution. Subsequently, the as-prepared PVDF solution was scraped on OICs, followed by heating at 90 °C for 30 min.

### 2.3. Temperature Tolerance Characterization

OICs were prepared in a rectangular parallelepiped with the dimensions of 9 cm × 8 cm × 2 mm. Thermogravimetric analysis (TGA) was performed on TGA-DSC1 (METTLER TOLEDO Co., shanghai, China) in the temperature range of 25 to 150 °C. The heating rate was 10 °C·min^−1^ and the analysis was carried out under N_2_ atmosphere. The dynamic thermomechanical analysis (DMA) was performed using Netzsch DMA242E (NETZSCH Co., Wittelsbacherstrasse, Germany) in the temperature range of −150 to −25 °C at a heating rate of 3 °C·min^−1^.

### 2.4. Impedance Analysis

OICs were clamped between two copper sheets with the same diameter and thickness and connected to the system by a wire. A Princeton Applied Research Versa STAT3 (AMETEK Co. Ltd, Shanghai, China) was used to analyze the impedance in the frequency range of 1 Hz to 100 kHz, with a temperature range of 0 to 75 °C and a bias voltage of 5 mV.

### 2.5. Mechanical Characterization

OICs were prepared in the form of a rectangular parallelepiped with the dimensions of 9 cm × 8 cm × 2 mm. The mechanical testing was performed on Tytron 250 (MTS Co., Eden Prairie MN, USA) at a rate of 100 mm·min^−1^. The initial distance was 20 mm.

### 2.6. Characterization of Self-Powered OICs

All OICs were stripped-off from the PET substrate before the verification test. Fourier transform infrared (FTIR) spectroscopy and X-ray diffraction (XRD) analysis were performed to evaluate the crystal structure of PVDF. The FTIR was performed using a Bruker VERTEX70 (city, the abbreviation of state, country Bruker Co., Billerica, MA, USA). The attenuated total reflection (ATR) mode was used, and the FTIR patterns were recorded in the wavelength range of 700 to 1500 cm^−1^. The XRD analysis was performed using Bruker D8 ADVANCE (city, the abbreviation of state, country Bruker Co., Billerica, MA, USA), and the XRD patterns were recorded in the 2θ range of 10 to 30°, and the wavelength was 0.154 nm. The piezoelectric constant d_33_ was measured using a quasi-static method on a d_33_ measurement instrument (ZJ-3AN) (Institute of Acoustics, Chinese Academy of Sciences, Beijing, China).

## 3. Results

**Figure 1:**
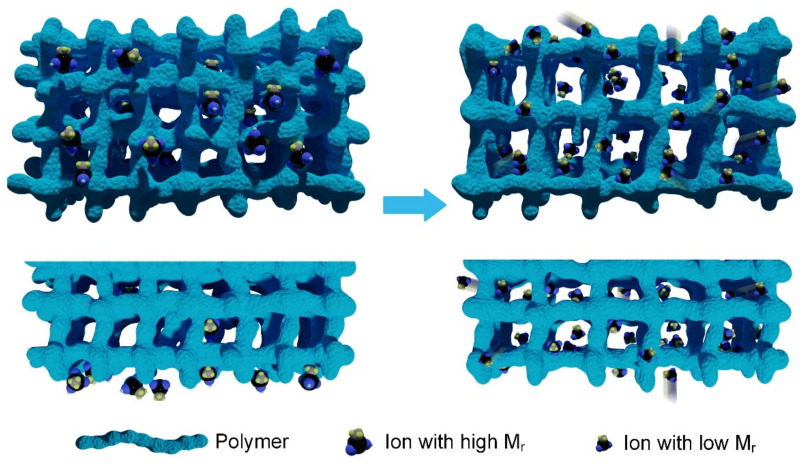
Schematic illustration of the proposed OICs.

We designed the OICs according to the migration of ions in electrolytes, where the high migration speed and ionic concentration were expected to render high conductivity. As shown in Figure 1, the ions possess a small relative molecular mass (Mr) and high solubility in PC, which can flow inside the OICs due to the rapid migration of the center of gravity [44]. However, the ions in the figure on the left only move at a slow speed, or even get stuck in the network, unable to move and restricting the movement of other ions. In addition, unlike the electrolyte solution, the staggered polymeric network limits the ionic movement in the gel, implying that the network structure and ionic size influence the ionic movement [45]. The network of the gel on the left is more complex and denser, and, although it may have slightly better mechanical properties, it becomes an obstacle to ion motion because the complex network reduces the passability of the road and reduces the flux of ions. Hence, we aimed to simplify the network and use small-sized molecules to seamlessly pass through the messy network (Figure 1).

**Figure 2:**
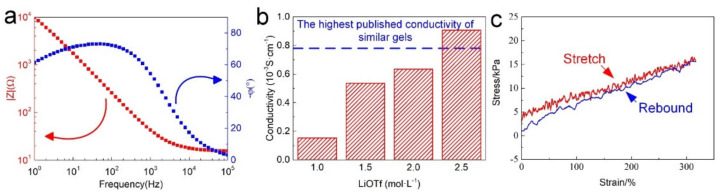
The electrical and mechanical properties of OICs: (**a**) frequency response of impedance magnitude (|Z|) and phase angle (φ), (**b**) conductivity of OICs at various LiOTf concentrations, (**c**) tensile and rebound curves of OICs.

OICs demonstrate remarkable ionic conductivity and decent mechanical properties. As presented in Figure 2a, the electrical characterization revealed a significant ionic conductivity with 2.5 mol·L^−1^ of LiOTf. With the frequency growth, the impedance amplitude linearly decreased until 1 kHz, obtaining an impedance value of 19.6 Ω and reached the minimum at 100 kHz (15.56 Ω). Meanwhile, the negative phase angle also exhibited a similar downtrend in the frequency range of 100 Hz to 100 kHz and sharply attenuated from 72° to a near-zero degree at 100 kHz. At this time, the influence of the electrical double layer was weak enough, and the impedance amplitude became close to a real resistance value. The conductivity of OICs was calculated to be 9.1 × 10^−4^ S·cm^−1^, exceeding the 7.9 × 10^−4^ S cm^−1^ of other similar types of organic ionic gels (Figure 2b). Surprisingly, the conductivity continuously climbed without any declining trend, contrary to that observed by other researchers, until the maximum solubility of LiOTf in PC, which indicates that the proposed composition significantly improved the saturation mobility of OICs. In addition, the stretch and recovery curves in Figure 2c show perfect overlap and minimal hysteresis until the strain reached 300%, indicating the superb tensile resilience of OICs. Consequently, unless otherwise specified, the LiOTf concentration in the OICs was fixed at 2.5 mol·L^−1^. Again, the salt with a relatively small size was shown to be more conducive to improving the conductivity of gels.

**Figure 3:**
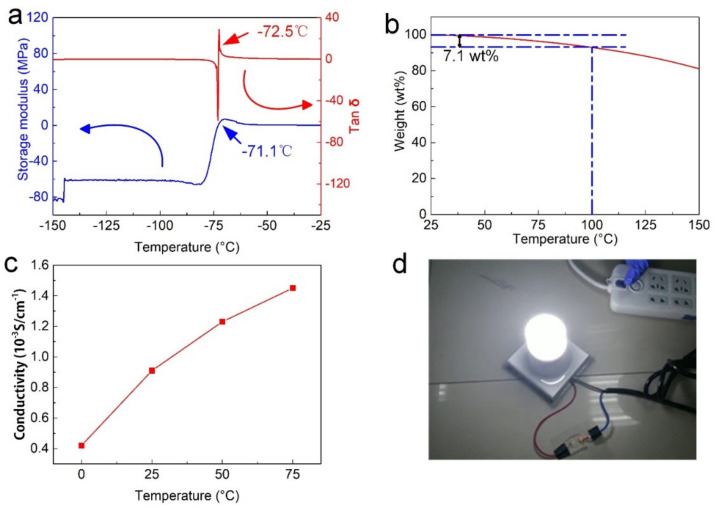
Thermal properties of OICs: (**a**) dynamic thermomechanical analysis and (**b**) thermogravimetric analysis. (**c**) Ionic conductivity of OICs in the temperature range of 0 to 75 °C, (**d**) lighting up a bulb using OICs instead of wires.

OICs exhibited extraordinary temperature tolerances due to the presence of PC. The dynamic thermomechanical analysis (Figure 3a) revealed that the glass-transition temperature of the OICs was about −70 °C, which remained stable at low temperatures. Furthermore, the smooth storage modulus curve reflects the uniform structure of OICs. The thermogravimetric analysis (Figure 3b) demonstrates that OICs are strikingly stable at high temperatures compared with hydrogels. Hence, we did not observe any significant weight loss at 50 °C and only a slight reduction of 7.1 wt.% was observed at 100 °C. More surprisingly, the solvent in OICs was not completely volatilized even at >100 °C, endowing outstanding temperature tolerance and ensuring the normal operation of OICs at high temperatures. Then, we analyzed the conductivity of OICs at different temperatures (Figure 3c). As expected, the increase in temperature (0 to 75 °C) accelerated the migration of ions that directly promoted the conductivity from 0.4 × 10^−3^ to 1.4 × 10^−3^ S·cm^−1^, respectively. Significantly, OICs presented an acceptable ionic conductivity over the entire temperature range, implying that OICs are suitable for most daily-life temperature conditions. Thus, the advantages of OICs in terms of temperature tolerance show that it is more competent for high- and low-temperature environments.

Finally, we further designed a circuit to visually display the ionic conductivity of OICs (Figure 3d). We replaced the wire with an OIC and successfully lit a household bulb (220 V). When the bulb was lit, no visible flicker was observed, and the brightness remained stable. Therefore, although the conductivity of OICs is not as outstanding as metallic conductors, OICs are still capable of replacing conventional wires.

**Figure 4:**
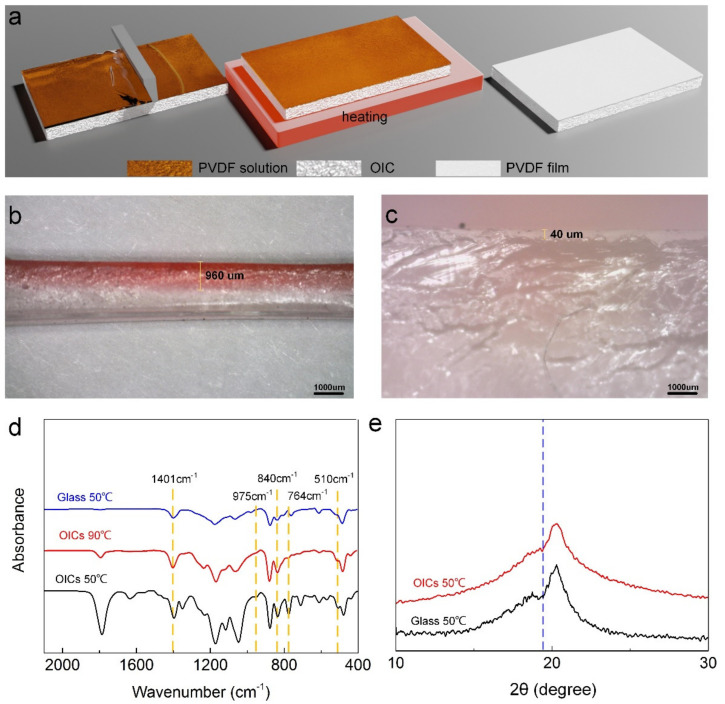
PVDF polarization detection. (**a**) Self-powered OIC preparation process, (**b**) penetration of DMF on OIC, and (**c**) the thickness of PVDF. (**d**) FTIR spectra of self-powered OICs on different substrates, and (**e**) XRD patterns of PVDF on glass and OICs after polarization.

Then, we covered OICs with PVDF to prepare a flexible conductor that could be self-powered. PVDF naturally crystallizes as a solvent, and this process is usually accompanied by heating. Suffering from easy evaporation of water, PVDF cannot be compounded with hydrogel, while OICs are different because of their extraordinary high-temperature resistance. Hence, self-powered OICs can be completed through a simple heating process (Figure 4a). Unlike dense and impervious substrates, such as glasses, the porous structure of OICs tends to absorb other solutions. In particular, DMF, as a selective organic solvent, can be used to dissolve PVDF and is perfectly miscible with the OIC precursors. As shown in Figure 4b, we added Sudan III for observation. DMF penetrates OIC to a depth of almost 1 mm after only 10 min of heating. Under the combined action of heat treatment, most of the DMF is lost, promoting the growth of PVDF crystals. At the same time, the liquid flows from PVDF solution to OIC reorient PVDF crystals to a certain extent, leading to a better polarization effect. Finally, a PVDF film about 40 μm thick was formed on the OIC (Figure 4c). As expected, PVDF cured on glass contained both α and β phases. Figure 4d presents the FTIR spectra of P-OICs, and we observed characteristic peaks for the α phase at 764 and 975 cm^−1^, and β/γ characteristic peaks were observed at 510, 840 and 1401 cm^−1^. When we heated PVDF on the OIC substrate at the same temperature, the intensity of each characteristic peak increased, indicating that the crystallinity of PVDF was significantly improved and therefore shows that better fluidity was beneficial to the formation of PVDF crystals. When we further increased the temperature to 90 °C, the α characteristic peaks at 764 and 975 cm^−1^ disappeared, which may have been due to the increased fluidity of DMF at higher temperatures, and the more intense directional flow of DMF promoting PVDF generation of the β-phase crystal form.

The peak intensity from the PVDF film on the OIC substrate was stronger than that on the glass substrate, according to which β-shaped crystals on OICs and glass were calculated to be 70.2% and 42.8%, respectively, thereby showing better polarization of PVDF on OICs. The XRD patterns were consistent with FTIR data (Figure 4e): a shoulder peak from the OIC substrate at 2θ = 20.6° was smoother than the glass substrate, indicating a relatively higher content of β-shaped crystals in OICs. Hence, crystallization is more effective on OICs. One should note that the direct heating of the organic gel to form the PVDF piezoelectric layer provides a more efficient and simple self-powered strategy for various flexible materials.

**Figure 5:**
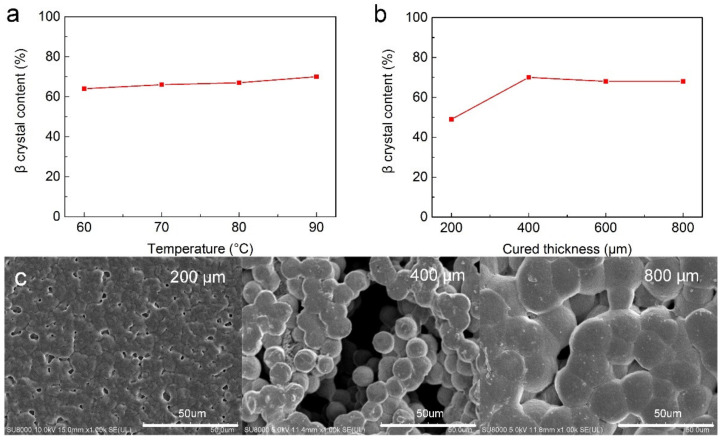
Polarization parameters of PVDF: β crystal content as a function of (**a**) temperature, (**b**) curing thickness, and (**c**) SEM images of PVDF layers with thicknesses of 200, 400 and 800 μm.

We further investigated the influence of heating temperature and thickness of the PVDF layer on polarization. As shown in Figure 5a, the content of β-shaped crystals rose slightly with increasing temperature. The higher temperature not only accelerated the crystallization but also intensified the thermal movement of molecules, yielding a slow upward trend of β-shaped crystal quantity. With increase in PVDF layer thickness, the grain size gradually increased. At the thicknesses of 200, 400 and 800 μm, the average diameters of the grains were 5, 10 and 25 μm, respectively. Simultaneously, we also discovered that the content of β-shaped crystals was raised when we applied 400 μm PVDF (Figure 5b). However, a further increase in PVDF layer thickness exerted no visual effect on the number of β-shaped crystals. As seen in SEM images (Figure 5c), the nearby molecules reunited when the layer thickness was 200 μm, preventing PVDF from forming spherical crystals, but simply covering it. However, when the squeegee thickness was raised to 800 μm, the spherical crystals continued to grow and even began to gradually merge, resulting in a low content of β-shaped crystals due to the overgrowth of grains. Hence, only a moderate thickness (400 μm) contributes to the desired crystalline state of PVDF.

Meanwhile, the observed spherical crystals generally represented a high β-shaped crystal content [46]. Thus, the piezoelectric effect of self-powered OICs was assessed using a 400 μm thick PVDF layer polarized at 90 °C. The piezoelectric coefficient of the conductor was found to be 15 pC·N^−1^, which is sufficient for OIC to work as a self-powered sensor.

**Figure 6:**
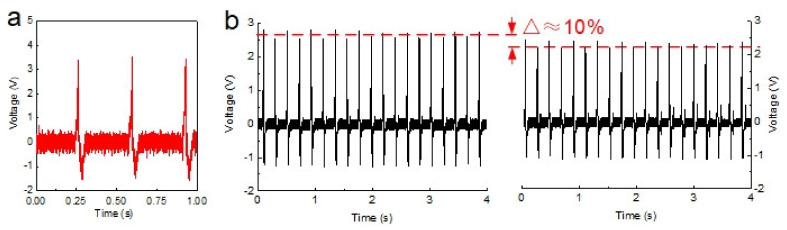
Piezoelectric characterization: (**a**) electromechanical response and (**b**) durability performance of self-powered OICs.

The electromechanical response shows excellent operating life of self-powered OICs. We performed the electromechanical test on the excitation platform at a frequency of 3 Hz and an excitation force of 36 N (Figure 6a). The self-powered OIC responded sensitively to the excitation force input and produced a stable voltage of 3.52 V, indicating that it already had piezoelectric characteristics. Additionally, the working life needed to be tested to prove its potential as a sensor. Consequently, a strict durability test was performed (Figure 6b), where we measured the output consumption under the frequency of 5 Hz and excitation force of 24 N before and after 10,000 excitation cycles and 30 days of room-temperature storage. The self-powered OICs stabilized the output voltage for a long time, and the loss rate was less than 10%, showing a long working life.

**Figure 7:**
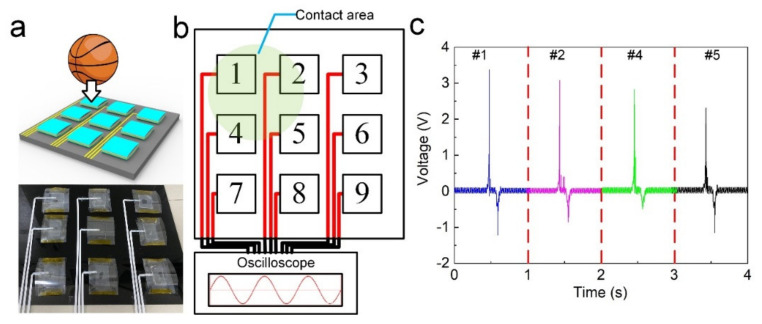
Application in basketball training: (**a**) the model and physical images of the basketball impact detection, (**b**) schematic diagram of the contact positions, and (**c**) the detected voltage.

Finally, we simulated the application of self-powered OICs in basketball training (Figure 7). The basketball impact was simulated on a platform, where nine sensors were arranged in a 3 × 3 matrix. According to the normal contact interval between two hits on the backboard, the excitation frequency was 1 Hz. When the impact occurred, the oscilloscope detected four signals indicating the hitting power and contact position, which can be used by coaches to train players.

## 4. Conclusions

In summary, we designed and manufactured highly conductive and self-powered OICs serving as self-powered sensors. The results revealed the ingenious choice of the small molecular weight lithium salt, which provided smooth ionic movement and enhanced the conductivity of OICs (9.1 × 10^−4^ S·cm^−1^). Moreover, benefiting from the excellent temperature tolerance within a range of −70 to 100 °C, we have combined the OIC with a PVDF piezoelectric layer to prepare self-powered OICs, achieving a stable voltage output and long working life. The OICs showed excellent potential for a wide range of applications in daily life as passive pressure sensors.

## Data Availability

The data are available upon reasonable request from the corresponding author.

## References

[1] Ohm Y., Pan C., Ford M.J., Huang X., Liao J., Majidi C. (2021). An electrically conductive silver–polyacrylamide–alginate hydrogel composite for soft electronics. Nat. Electron..

[2] Yuk H., Lu B., Zhao X. (2019). Hydrogel bioelectronics. Chem. Soc. Rev..

[3] Zhang Z.X., Wang L., Yu H.T., Zhang F., Tang L., Feng Y.Y., Feng W. (2020). Highly Transparent, Self-Healable, and Adhesive Organogels for Bio-Inspired Intelligent Ionic Skins. ACS Appl. Mater. Interfaces.

[4] Zhu J., Li F.X., Wang X.L., Yu J.Y., Wu D.Q. (2018). Hyaluronic Acid and Polyethylene Glycol Hybrid Hydrogel Encapsulating Nanogel with Hemostasis and Sustainable Antibacterial Property for Wound Healing. ACS Appl. Mater. Interfaces.

[5] Oh Y.S., Kim J.H., Xie Z.Q., Cho S., Han H., Jeon S.W., Park M., Namkoong M., Avila R., Song Z. (2021). Battery-free, wireless soft sensors for continuous multi-site measurements of pressure and temperature from patients at risk for pressure injuries. Nat. Commun..

[6] Xu W., Huang L.B., Wong M.C., Chen L., Bai G.X., Hao J.H. (2017). Environmentally Friendly Hydrogel-Based Triboelectric Nanogenerators for Versatile Energy Harvesting and Self-Powered Sensors. Adv. Energy Mater..

[7] Sahu M., Vivekananthan V., Hajra S., Khatua D.K., Kim S.J. (2021). Porosity modulated piezo-triboelectric hybridized nanogenerator for sensing small energy impacts. Appl. Mater. Today.

[8] Gao G., Yang F., Zhou F., He J., Lu W., Xiao P., Yan H., Pan C., Chen T., Wang Z.L. (2020). Bioinspired Self-Healing Human–Machine Interactive Touch Pad with Pressure-Sensitive Adhesiveness on Targeted Substrates. Adv. Mater..

[9] Wang Z., Cong Y., Fu J. (2020). Stretchable and tough conductive hydrogels for flexible pressure and strain sensors. J. Mater. Chem. B.

[10] Liu X., Liu J., Lin S., Zhao X. (2020). Hydrogel machines. Mater. Today.

[11] Yang C., Suo Z. (2018). Hydrogel ionotronics. Nat. Rev. Mater..

[12] Wang C., Zhang P., Xiao W., Zhao J., Shi M., Wei H., Deng Z., Guo B., Zheng Z., Yu Y. (2020). Visible-light-assisted multimechanism design for one-step engineering tough hydrogels in seconds. Nat. Commun..

[13] Chun S., Choi I.Y., Son W., Jung J., Lee S., Kim H.S., Pang C., Park W., Kim J.K. (2019). High-Output and Bending-Tolerant Triboelectric Nanogenerator Based on an Interlocked Array of Surface-Functionalized Indium Tin Oxide Nanohelixes. ACS Energy Lett..

[14] Qi J.B., Wang A.C., Yang W.F., Zhang M.Y., Hou C.Y., Zhang Q.H., Li Y.G., Wang H.Z. (2020). Hydrogel-based hierarchically wrinkled stretchable nanofibrous membrane for high performance wearable triboelectric nanogenerator. Nano Energy.

[15] De Medeiros M.S., Chanci D., Martinez R.V. (2020). Moisture-insensitive, self-powered paper-based flexible electronics. Nano Energy.

[16] Rahimi A., Herzog-Arbeitman A., García J.M. (2019). Conductive Recyclable Organogel Composites. Macromol. Mater. Eng..

[17] Cai J., Zhang X., Liu W., Huang J., Qiu X. (2020). Synthesis of highly conductive hydrogel with high strength and super toughness. Polymer.

[18] Chun K.Y., Seo S., Han C.S. (2021). Self-Powered, Stretchable, and Wearable Ion Gel Mechanoreceptor Sensors. ACS Sens..

[19] Ji Z., Yan C., Yu B., Zhang X., Cai M., Jia X., Wang X., Zhou F. (2019). 3D Printing of Hydrogel Architectures with Complex and Controllable Shape Deformation. Adv. Mater. Technol..

[20] Kazunari Y., Yuki T., Yuta H., Masaru K., Hidemitsu F. 3D printing for gel robotics. Proceedings of the SPIE Smart Structures and Materials + Nondestructive Evaluation and Health Monitoring.

[21] Schroeder T.B.H., Guha A., Lamoureux A., VanRenterghem G., Sept D., Shtein M., Yang J., Mayer M. (2017). An electric-eel-inspired soft power source from stacked hydrogels. Nature.

[22] Hao X.P., Li C.Y., Zhang C.W., Du M., Ying Z.M., Zheng Q., Wu Z.L. (2021). Self-Shaping Soft Electronics Based on Patterned Hydrogel with Stencil-Printed Liquid Metal. Adv. Funct. Mater..

[23] Li G.X., Li L.W., Zhang P.P., Chang C.Y., Xu F., Pu X. (2021). Ultra-stretchable and healable hydrogel-based triboelectric nanogenerators for energy harvesting and self-powered sensing. RSC Adv..

[24] Wang S.H., Wang Z.L., Yang Y. (2016). A One-Structure-Based Hybridized Nanogenerator for Scavenging Mechanical and Thermal Energies by Triboelectric-Piezoelectric-Pyroelectric Effects. Adv. Mater..

[25] Zhang H.X., Niu W.B., Zhang S.F. (2018). Extremely Stretchable, Stable, and Durable Strain Sensors Based on Double-Network Organogels. ACS Appl. Mater. Interfaces.

[26] Ahmed K., Naga N., Kawakami M., Furukawa H. (2018). Extremely Soft, Conductive, and Transparent Ionic Gels by 3D Optical Printing. Macromol. Chem. Phys..

[27] Li C., Cong S., Tian Z.N., Song Y.Z., Yu L.H., Lu C., Shao Y.L., Li J., Zou G.F., Rummeli M.H. (2019). Flexible perovskite solar cell-driven photo-rechargeable lithium-ion capacitor for self-powered wearable strain sensors. Nano Energy.

[28] Li Z., Liu Z., Ng T.Y., Sharma P. (2020). The effect of water content on the elastic modulus and fracture energy of hydrogel. Extrem. Mech. Lett..

[29] Ming Z.Z., Pang Y., Liu J.Y. (2020). Switching between Elasticity and Plasticity by Network Strength Competition. Adv. Mater..

[30] Rahman M.S., Shiblee M.D.N.I., Ahmed K., Khosla A., Ogawa J., Kawakami M., Furukawa H. (2020). Flexible and Conductive 3D Printable Polyvinylidene Fluoride and Poly(N,N-dimethylacrylamide) Based Gel Polymer Electrolytes. Macromol. Mater. Eng..

[31] Alam A., Moussa M. (2020). Preparation of graphene/poly(vinyl alcohol) composite hydrogel films with enhanced electrical and mechanical properties. Polym. Compos..

[32] Liu X., Miller A.L., Park S., Waletzki B.E., Zhou Z., Terzic A., Lu L. (2017). Functionalized Carbon Nanotube and Graphene Oxide Embedded Electrically Conductive Hydrogel Synergistically Stimulates Nerve Cell Differentiation. ACS Appl. Mater. Interfaces.

[33] Han S., Liu C., Lin X., Zheng J., Wu J., Liu C. (2020). Dual Conductive Network Hydrogel for a Highly Conductive, Self-Healing, Anti-Freezing, and Non-Drying Strain Sensor. ACS Appl. Polym. Mater..

[34] Wu L., Hu Y., Tang P., Wang H., Bin Y. (2021). High stretchable, pH-sensitive and self-adhesive rGO/CMCNa/PAA composite conductive hydrogel with good strain-sensing performance. Compos. Commun..

[35] Yang Y.Y., Yang Y.T., Cao Y.X., Wang X., Chen Y.R., Liu H.Y., Gao Y.F., Wang J.F., Liu C., Wang W.J. (2021). Anti-freezing, resilient and tough hydrogels for sensitive and large-range strain and pressure sensors. Chem. Eng. J..

[36] Chen H., Huang J., Liu J., Gu J., Zhu J., Huang B., Bai J., Guo J., Yang X., Guan L. (2021). High toughness multifunctional organic hydrogels for flexible strain and temperature sensor. J. Mater. Chem. A.

[37] Zhu T., Jiang C., Wang M.L., Zhu C.Z., Zhao N., Xu J. (2021). Skin-Inspired Double-Hydrophobic-Coating Encapsulated Hydrogels with Enhanced Water Retention Capacity. Adv. Funct. Mater..

[38] Xu R.D., Qu L.J., Tian M.W. (2021). Touch-sensing fabric encapsulated with hydrogel for human-computer interaction. Soft Matter.

[39] Jian Y., Handschuh-Wang S., Zhang J., Lu W., Zhou X., Chen T. (2021). Biomimetic anti-freezing polymeric hydrogels: Keeping soft-wet materials active in cold environments. Mater. Horiz..

[40] Sui X., Guo H., Chen P., Zhu Y., Wen C., Gao Y., Yang J., Zhang X., Zhang L. (2020). Zwitterionic Osmolyte-Based Hydrogels with Antifreezing Property, High Conductivity, and Stable Flexibility at Subzero Temperature. Adv. Funct. Mater..

[41] Subraveti S.N., Raghavan S.R. (2021). A Simple Way to Synthesize a Protective “Skin” around Any Hydrogel. ACS Appl. Mater. Interfaces.

[42] Gao Y., Shi L., Lu S., Zhu T., Da X., Li Y., Bu H., Gao G., Ding S. (2019). Highly Stretchable Organogel Ionic Conductors with Extreme-Temperature Tolerance. Chem. Mater..

[43] Sun H., Zhao Y., Jiao S., Wang C., Jia Y., Dai K., Zheng G., Liu C., Wan P., Shen C. (2021). Environment Tolerant Conductive Nanocomposite Organohydrogels as Flexible Strain Sensors and Power Sources for Sustainable Electronics. Adv. Funct. Mater..

[44] Lee Y.Y., Kang H.Y., Gwon S.H., Choi G.M., Lim S.M., Sun J.Y., Joo Y.C. (2016). A Strain-Insensitive Stretchable Electronic Conductor: PEDOT:PSS/Acrylamide Organogels. Adv. Mater..

[45] Ueda C., Park J., Hirose K., Konishi S., Ikemoto Y., Osaki M., Yamaguchi H., Harada A., Tanaka M., Watanabe G. (2022). Behavior of supramolecular cross-links formed by host-guest interactions in hydrogels responding to water contents. Supramol. Mater..

[46] Fu W.L., Du P.Y., Weng W.J., Han G.Y. (2005). Preparation and structure of PVDF piezoelectric film. Chin. J. Mater. Res..

